# Determinants and Impact of *Giardia* Infection in the First 2 Years of Life in the MAL-ED Birth Cohort

**DOI:** 10.1093/jpids/piw082

**Published:** 2017-02-15

**Authors:** Elizabeth T. Rogawski, Luther A. Bartelt, James A. Platts-Mills, Jessica C. Seidman, Amidou Samie, Alexandre Havt, Sudhir Babji, Dixner Rengifo Trigoso, Shahida Qureshi, Sadia Shakoor, Rashidul Haque, Estomih Mduma, Samita Bajracharya, S. M. Abdul Gaffar, Aldo A. M. Lima, Gagandeep Kang, Margaret N. Kosek, Tahmeed Ahmed, Erling Svensen, Carl Mason, Zulfiqar A. Bhutta, Dennis R. Lang, Michael Gottlieb, Richard L. Guerrant, Eric R. Houpt, Pascal O. Bessong, the MAL-ED Network Investigators

**Affiliations:** 1 Division of Infectious Diseases and International Health, University of Virginia, Charlottesville;; 2 Division of Infectious Diseases, University of North Carolina-Chapel Hill;; 3 Fogarty International Center, National Institutes of Health, Bethesda, Maryland;; 4 University of Venda, Thohoyandou, South Africa;; 5 Clinical Research Unit and Institute of Biomedicine, Federal University of Ceara, Fortaleza, Brazil;; 6 Christian Medical College, Vellore, India;; 7 Asociación Benéfica PRISMA, Iquitos, Peru;; 8 Aga Khan University, Karachi, Pakistan;; 9 International Centre for Diarrhoeal Disease Research, Dhaka, Bangladesh;; 10 Haydom Lutheran Hospital, Haydom, Tanzania;; 11 Walter Reed AFRIMS Research Unit Nepal, Kathmandu, Nepal;; 12 Bloomberg School of Public Health, Johns Hopkins University, Baltimore, Maryland;; 13 Haukeland University Hospital, Bergen, Norway;; 14 Armed Forces Research Institute of Medical Sciences, Bangkok, Thailand; and; 15 Foundation for the National Institutes of Health, Bethesda, Maryland

**Keywords:** children, Giardia, growth, intestinal permeability, risk factors.

## Abstract

**Background.:**

*Giardia* are among the most common enteropathogens detected in children in low-resource settings. We describe here the epidemiology of infection with *Giardia* in the first 2 years of life in the Etiology, Risk Factors, and Interactions of Enteric Infections and Malnutrition and the Consequences for Child Health and Development Project (MAL-ED), a multisite birth-cohort study.

**Methods.:**

From 2089 children, 34916 stool samples collected during monthly surveillance and episodes of diarrhea were tested for *Giardia* using an enzyme immunoassay. We quantified the risk of *Giardia* detection, identified risk factors, and assessed the associations with micronutrients, markers of gut inflammation and permeability, diarrhea, and growth using multivariable linear regression.

**Results.:**

The incidence of at least 1 *Giardia* detection varied according to site (range, 37.7%–96.4%) and was higher in the second year of life. Exclusive breastfeeding (HR for first *Giardia* detection in a monthly surveillance stool sample, 0.46 [95% confidence interval (CI), 0.28–0.75]), higher socioeconomic status (HR, 0.74 [95% CI, 0.56–0.97]), and recent metronidazole treatment (risk ratio for any surveillance stool detection, 0.69 [95% CI, 0.56–0.84]) were protective. Persistence of *Giardia* (consecutive detections) in the first 6 months of life was associated with reduced subsequent diarrheal rates in Naushahro Feroze, Pakistan but not at any other site. *Giardia* detection was also associated with an increased lactulose/mannitol ratio. Persistence of *Giardia* before 6 months of age was associated with a −0.29 (95% CI, −0.53 to −0.05) deficit in weight-for-age z score and −0.29 (95% CI, −0.64 to 0.07) deficit in length-for-age z score at 2 years.

**Conclusions.:**

Infection with *Giardia* occurred across epidemiological contexts, and repeated detections in 40% of the children suggest that persistent infections were common. Early persistent infection with *Giardia*, independent of diarrhea, might contribute to intestinal permeability and stunted growth.

## Summary

In a multisite birth-cohort study, *Giardia* spp were detected by enzyme immunoassay at least once in two-thirds of the children. Early persistent infection with *Giardia*, independent of diarrhea, was associated with deficits in both weight and length at 2 years of age.

## INTRODUCTION

*Giardia lamblia*, also known as *Giardia duodenalis* and *Giardia intestinalis*, is the most common etiology of intestinal parasitic infection in the first 2 years of life in low-resource settings. Although *Giardia* is a recognized pathogen of waterborne diarrhea outbreaks [1] and a common cause of diarrhea among travelers [2–4] and after recreational water exposure [5], the impact of endemic pediatric giardiasis is less clear. Two large studies of global etiologies of endemic pediatric diarrhea, the Global Enterics Multicenter Study (GEMS) [6] and the Etiology, Risk Factors, and Interactions of Enteric Infections and Malnutrition and the Consequences for Child Health and Development Project (MAL-ED) [7], found *Giardia* significantly more often in nondiarrheal than diarrheal stools. Similarly, *Giardia* was not associated with acute diarrhea in a meta-analysis of 12 acute pediatric diarrhea studies [4], and *Giardia* had a protective effect against acute diarrhea in 2 longitudinal studies [8, 9].

Evidence for an association between *Giardia* infection and child growth outcomes has been mixed [10–15]. *Giardia* infection is associated with disrupted villus architecture [16], an elevated lactulose/mannitol ratio (a marker of intestinal permeability) [17, 18], and zinc and vitamin A deficiencies [19–21], which suggests gut dysfunction and inadequate nutrient uptake. These associations, however, have been inconsistent and limited in ascribing directionality, because vitamin A deficiency, for example, can increase susceptibility to *Giardia* infection [22].

The multisite MAL-ED birth-cohort study [23] provides high-resolution prospective data to clarify early-life *Giardia* epidemiology in high-prevalence settings. Longitudinal analysis specifically enables assessment of the temporality between detection of *Giardia*, diarrhea, micronutrient status, markers of intestinal permeability and inflammation, and the estimation of longer-term effects on growth. Here, we describe the determinants, burden, and impact of *Giardia* infection in the first 2 years of life in 8 low-resource sites.

## METHODS

The MAL-ED study design and methods have been described [23]. In brief, the study was conducted between November 2009 and February 2014 at sites in Dhaka, Bangladesh (BGD), Fortaleza, Brazil (BRF), Vellore, India (INV), Bhaktapur, Nepal (NEB), Naushahro Feroze, Pakistan (PKN), Loreto, Peru (PEL), Venda, South Africa (SAV), and Haydom, Tanzania (TZH). Children were followed from birth (<17 days of age) via twice-weekly home visits for illness surveillance, medicines, and breastfeeding practices and monthly for anthropometry until they reached 2 years of age [24]. Nondiarrheal surveillance stool samples were collected and tested for 40 enteropathogens [25] monthly in the first year (0–12 months) of life and quarterly in the second year (12–24 months) of life. Stool samples were collected and tested also during every diarrhea episode reported during the twice-weekly surveillance visits. Diarrhea was defined as maternal report of 3 or more loose stools in 24 hours or 1 stool with visible blood [24]. Weight-for-age (WAZ) and length-for-age (LAZ) z scores were calculated using the 2006 World Health Organization child growth standards [26]. Sociodemographic information was assessed biannually and summarized using the Water, Assets, Maternal Education, Income (WAMI) score, which is based on monthly household income, maternal education, wealth measured by 8 assets, and access to improved water and sanitation [27], as defined by World Health Organization guidelines [28]. Plasma zinc and retinol concentrations were assessed at 7, 15, and 24 months of age [29]. Urinary lactulose/mannitol excretion ratios, measured at 3, 6, 9, and 15 months of age, were converted into sample-based z scores (LMZs) using the BRF cohort as the internal reference population [30]. All sites received ethical approval from their respective governmental, local institutional, and collaborating institutional ethical review boards. Informed written consent was obtained from the parent or guardian of each child.

### Data and Definitions

We included in the analysis all monthly surveillance and diarrheal stool samples that were tested for *Giardia* by enzyme immunoassay (EIA) (TechLab, Blacksburg, VA), the majority of which were also tested by wet-prep microscopy. The laboratory methods for detecting other enteropathogens and gut biomarkers, including α-1-antitrypsin (ALA), myeloperoxidase (MPO), neopterin (NEO), and α-1-acid glycoprotein (AGP), a marker of systemic inflammation, have been described [25, 29, 31].

Definitions of incident *Giardia*-related diarrhea were defined with increasing specificity for diarrhea of true *Giardia* etiology as follows: (1) *Giardia*-positive diarrhea, *Giardia* was detected in a diarrheal stool sample; (2) new *Giardia*-positive diarrhea, *Giardia* was detected in a diarrheal stool sample, and the most recent previous stool sample tested negative for *Giardia* or was taken more than 2 months earlier; (3) *Giardia*-positive diarrhea-associated pathogens–negative diarrhea, *Giardia* was detected in a diarrheal stool sample, but no diarrhea-associated pathogens that were previously identified in MAL-ED were detected (13 of 40 pathogens tested, ie, norovirus GII, rotavirus, astrovirus, adenovirus, *Campylobacter*, *Cryptosporidium*, heat-stable enterotoxin-producing enterotoxigenic *Escherichia coli*, typical enteropathogenic *E coli*, heat-labile enterotoxin-producing enterotoxigenic *E coli*, *Shigella*, enteroinvasive *E coli*, *Entamoeba histolytica*, and *Salmonella* [7]); and (4) *Giardia*-positive-only diarrhea, *Giardia* was detected in a diarrheal stool sample, and no other enteropathogens among all 40 tested were detected [25]. Persistence of *Giardia* detection was defined as 2 consecutive stool samples that tested positive for *Giardia* (2 consecutive months in the first year of life or 2 consecutive quarters in the second year). Prolonged persistence was defined as 3 consecutive stool samples that tested positive for *Giardia*.

### Data Analysis

Risk factors for the first detection of *Giardia* in surveillance stool samples were identified using pooled logistic regression to estimate hazard ratios (HRs) and adjusting for site and a restricted quadratic spline [32] for age. Variables in the multivariable model were included on the basis of statistical significance, model fit by the quasi-likelihood information criterion, covariance between factors, and variability of factors within sites for site-specific models. Comparing by the Akaike information criterion (AIC) to models with linear week of the year, seasonality was assessed by modeling *Giardia* detection with linear, quadratic, and cubic terms for the week of the year (*w*), and the terms sin(2π*w*/52), cos(2π*w*/52), sin(4π*w*/52), and cos(4π*w*/52). We used Poisson regression to evaluate associations between zinc and vitamin A status with *Giardia* detection in surveillance stool samples and adjusted for previous *Giardia* detection and potential confounders included in the multivariable risk factor model. We estimated the effect of *Giardia* detection on subsequent diarrheal rates using pooled logistic regression with general estimating equations (GEEs) and robust variance to account for correlation between outcomes within children and adjusted for the same confounders and illness symptoms during the exposure periods. We estimated the effect of *Giardia* in all stools on gut biomarker concentrations using multivariable linear regression with GEEs and adjusted for stool consistency and presence of the 2 other pathogens of highest prevalence, enteroaggregative *E coli* (EAEC) and *Campylobacter*. Last, we estimated the effect of *Giardia* detection in surveillance stools on WAZ and LAZ attainment at 2 years of age using multivariable linear regression with GEEs. Confounders, listed in the table footnotes, included baseline sociodemographic characteristics associated with *Giardia* detection identified above and EAEC and *Campylobacter* stool positivity. Data from SAV were excluded from zinc-related analyses and data from PKN were excluded from length-related analyses because of measurement quality concerns at those sites. For analyses limited to surveillance stool samples, results (not shown) were consistent when we repeated analyses with diarrheal stool samples.

## RESULTS

### Diagnostics

Of 34916 stool samples (27092 surveillance and 7824 diarrheal) tested for *Giardia* by an EIA, 33796 (96.8%) were also tested for *Giardia* by wet-prep microscopy. Compared to EIA, the sensitivity of microscopy was 46.2%, and its specificity was 99.3%. *Giardia* positivity by microscopy was 21% less likely if the stool was watery or liquid than if it was soft or formed (risk ratio [RR], 0.79 [95% confidence interval (CI), 0.68–0.90]), but we found no association between stool consistency and EIA results (RR, 0.94 [95% CI, 0.86–1.03]).

### Incidence and Persistence

**Figure 1.**
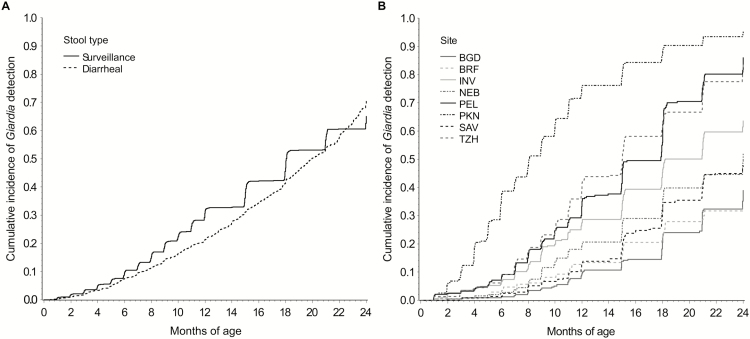
Cumulative incidence of *Giardia* detection in surveillance and diarrheal stool samples across all sites (A) and in surveillance stool samples within each site (B) among 2089 children in the Etiology, Risk Factors, and Interactions of Enteric Infections and Malnutrition and the Consequences for Child Health and Development Project (MAL-ED) cohort with at least 1 stool tested for *Giardia*.

Among 2089 children with at least 1 tested stool, the overall *Giardia* prevalence according to the EIA in stool samples was 14.7% (n = 5135). *Giardia* was detected at least once in two-thirds (n = 1178) of the 1741 children followed to 2 years of age (range, 37.7% [BRF] to 96.4% [PKN]). The overall median times to *Giardia* detection, which varied according to site, were 18.0 and 20.0 months for surveillance and diarrheal stool samples, respectively (Figure 1).

The incidence of *Giardia*-positive diarrhea was 40.4 cases per 100 person-years. However, measures of *Giardia*-related diarrhea incidence decreased by approximately 60%, 75%, and more than 80% when we required the previous stool sample to have tested negative for *Giardia*, the current stool sample to have no detection of diarrhea-associated pathogens, and the current stool sample to have no detection of any other pathogens, respectively (Table 1).

**Table 1.** Prevalence of *Giardia* Detection in Surveillance Stools and Incidence of *Giardia*-Related Diarrhea According to Site Among 2089 Children in the MAL-ED Birth Cohort

Site	Prevalence (%) of *Giardia* Detection in Monthly Surveillance Stools	Incidence^a^ of *Giardia*- Positive Diarrhea	Incidence^a^ of New *Giardia*- Positive Diarrhea^b^	Incidence^a^ of *Giardia*-Positive Diarrhea- Associated Pathogens–Negative Diarrhea^c^	Incidence^a^ of *Giardia*-Positive- Only Diarrhea^d^
BGD	4.2	22.8 (18.7–27.7)	12.3 (9.4–16.1)	1.8 (0.9–3.6)	1.4 (0.6–3.0)
BRF	7.3	4.6 (2.9–7.4)	1.8 (0.9–3.8)	1.5 (0.7–3.4)	1.0 (0.4–2.8)
INV	13.1	21.4 (17.6–26.1)	8.1 (5.9–11.2)	6.6 (4.6–9.4)	4.4 (2.8–6.8)
NEB	9.2	18.2 (14.6–22.6)	7.5 (5.4–10.5)	6.6 (4.6–9.5)	5.1 (3.4–7.7)
PEL	16.4	115.8 (106.3–126.2)	40.4 (34.9–46.7)	28.7 (24.1–34.0)	18.7 (15.1–23.2)
PKN	35.2	126.2 (116.3–136.9)	42.5 (36.9–48.9)	28.3 (23.8–33.6)	15.7 (12.4–19.7)
SAV	6.4	4.1 (2.6–6.3)	2.4 (1.4–4.3)	1.0 (0.4–2.4)	0.61 (0.20–1.9)
TZH	16.2	7.0 (5.0–9.9)	3.7 (2.3–6.0)	0.44 (0.11–1.8)	0.22 (0.03–1.6)
All	13.6	40.4 (38.4–42.5)	15.0 (13.8–16.3)	9.5 (8.5–10.5)	5.9 (5.2–6.8)

Abbreviations: BGD, Dhaka, Bangladesh; BRF, Fortaleza, Brazil; INV, Vellore, India; NEB, Bhaktapur, Nepal; PEL, Loreto, Peru; PKN, Naushahro Feroze, Pakistan; SAV, Venda, South Africa; TZH, Haydom, Tanzania.

^a^Rate per 100 person-years.

^b^New *Giardia*-positive diarrhea was defined if *Giardia* was detected in the diarrheal stool and the most recent previous stool tested negative for *Giardia* or was taken more than 2 months earlier (n = 539 of 1452 [37.1%] of all *Giardia*-positive diarrheal stools).

^c^ *Giardia*-positive diarrhea-associated pathogens–negative diarrhea was defined if *Giardia* was detected in the diarrheal stool and no diarrhea-associated pathogens were detected (n = 341 of 1291 [26.4%] of all completely tested *Giardia*-positive diarrheal stools). Diarrhea-associated pathogens (norovirus GII, rotavirus, astrovirus, adenovirus, *Campylobacter*, *Cryptosporidium*, heat-stable enterotoxin-producing enterotoxigenic *E coli*, typical enteropathogenic *E coli*, heat-labile enterotoxin-producing enterotoxigenic *E coli*, *Shigella*, enteroinvasive *E coli*, *E. histolytica*, and *Salmonella*) were associated with diarrhea in the first or second year of life [7].

^d^ *Giardia*-positive-only diarrhea was defined if *Giardia* was detected in the diarrheal stool and no other pathogens were detected (n = 214 of 1291 [16.6%] of all completely tested *Giardia*-positive diarrheal stool samples).

The overall prevalence of *Giardia* detected in surveillance stool samples was 13.6% (Table 1). However, the prevalence decreased by more than half (6.0%) when we required the previous surveillance stool to have tested negative for *Giardia*. Repeated *Giardia* detections in surveillance stool samples occurred in 838 (40.1%) children (Supplementary Figure 1). The prevalence of persistence was less than 5% before 6 months of age in all except the PKN site but increased to 31.8% overall in the second year of life.

### Risk Factors

*Giardia* detection increased with age over the first 2 years of life; a 1-month increase in age was associated with an 11% increase in the risk of *Giardia* detection in surveillance stool samples (RR, 1.11 [95% CI, 1.10–1.12]). The percentage of days in the previous month that the child was exclusively breastfed was a strongly protective factor against first *Giardia* detection (Table 2). Socioeconomic factors, including increased socioeconomic score (Water, Assets, Maternal Education, Income score [27]), household income, and older maternal age, were also protective. Metronidazole exposure in the previous 15 days was associated with a 31% relative decrease (95% CI, 16–44) in *Giardia* detection in surveillance stool samples, but we found no association with exposure more than 15 days earlier or with exposure to any other antibiotics.

**Table 2.** Risk Factors for First *Giardia* Detection in Monthly Surveillance Stool Samples Among 2088 Children in the MAL-ED Cohort With at Least 1 Surveillance Stool

Risk Factor	Semi-Univariable (HR [95% CI])^a^	Multivariable (HR [95% CI])^b^
Child characteristics		
Female (vs male)	1.02 (0.89–1.16)	
Enrollment weight (per 1 z score)	1.05 (0.98–1.12)	
Exclusively breastfed since birth	0.67 (0.37–1.20)	
Percent days exclusively breastfed in last month (100% vs 0%)	0.43 (0.27–0.70)	0.46 (0.28–0.75)
WAZ (per 1 z score)^c^	0.94 (0.88–1.00)	0.97 (0.91–1.03)
LAZ (per 1 z score)^c^,^d^	0.96 (0.89–1.03)	
WLZ (per 1 z score)^c,d^	0.94 (0.88–1.00)	
Metronidazole used in previous 15 days	0.69 (0.47–1.01)	0.71 (0.48–1.04)
Metronidazole used in previous 16–30 days	1.02 (0.73–1.42)	
Sociodemographic		
Socioeconomic score (WAMI [27], per 0.5 unit)	0.54 (0.42–0.69)	0.74 (0.56–0.97)
Household income at or above site-specific median income	0.79 (0.69–0.91)	
Maternal age (per 5 y)	0.83 (0.75–0.93)	0.86 (0.77–0.96)
Maternal education (≥6 y completed)	0.74 (0.64–0.86)	
Mother married	1.24 (0.95–1.60)	
Child has siblings	1.36 (1.18–1.57)	1.26 (1.09–1.46)
Mean no. of people per room in the household (per 1 unit)	1.09 (1.04–1.16)	
Water		
Improved (vs unimproved) drinking water [28]	0.95 (0.72–1.25)	
Time to access water (>10 min)	1.18 (0.97–1.44)	
Treated (vs untreated) water	0.69 (0.54–0.88)	0.78 (0.61–1.00)
Sanitation and hygiene		
Improved (vs unimproved) sanitation [28]	0.89 (0.71–1.10)	
Always washed hands after child defecated	0.79 (0.71–0.89)	0.80 (0.68–0.95)
Always washed hands before preparing food	0.85 (0.77–0.95)	
Always washed hands after using toilet	0.82 (0.73–0.92)	
Used toilet paper	0.86 (0.76–0.98)	0.95 (0.83–1.08)
Shared toilet facility	1.10 (0.89–1.35)	
Environmental		
Dirt floor	1.34 (1.10–1.62)	1.13 (0.92–1.38)
Household owned cows	1.04 (0.80–1.35)	
Household owned chickens	1.38 (1.12–1.70)	1.35 (1.10–1.66)

Abbreviations: CI, confidence interval; HR, hazard ratio; LAZ, length-for-age z score; WAMI, Water, Assets, Maternal Education, Income; WAZ, weight-for-age z score; WLZ, weight-for-length z score.

^a^Adjusted for site and age only (using restricted quadratic splines).

^b^Adjusted for site, age, and all other variables with estimates in this column (significant or strong association with *Giardia* detection in semi-univariable analysis, and not collinear with other variables).

^c^At most recent measurement before stool collection.

^d^Excluding PKN.

Multiple hygiene and environmental risk factors were associated with *Giardia* detection. Hand-washing, treatment of drinking water, and increased water access were protective, whereas the presence of siblings was a strong risk factor. Associations with having a dirt floor and owning chickens indicate the importance of environmental exposure to *Giardia* (Table 2). The distribution of environmental factors differed according to site, and although risk factor trends were generally consistent, there were site-to-site variations in the magnitude and even the direction of associations in some cases (Supplementary Figure 2).

*Giardia* was positively correlated with *Campylobacter* detection (Pearson correlation coefficient [PCC], 0.15; *P* < .0001) but not with the detection of EAEC (PCC, −0.02; *P* = .0001) or viruses (PCC, −0.00; *P* = .7) in all stool samples, which suggests that the routes of transmission and/or age-susceptibility patterns are similar to those of *Campylobacter*.

### Seasonality

We found a significant increase in first *Giardia* detections in surveillance stool samples in July through September and a smaller peak in March/April in the south Asian sites (BGD, INV, NEB, and PKN) (Supplementary Figure 3). *Giardia* seasonality was variable at the other sites, with peaks in December/January in the BRF and PEL sites and a small peak in March/April in the TZH and SAV sites. In contrast, we found no evidence of seasonality when we included all *Giardia* detections. No association between site-specific mean temperature or rainfall and *Giardia* positivity was found. The seasonality of *Giardia* detection in diarrheal stool samples matched the seasonality of all-cause diarrhea, which suggests that many *Giardia*-positive diarrheal episodes were caused by other pathogens (not shown).

### Zinc, Vitamin A, and *Giardia*

Higher plasma zinc and retinol concentrations at 7 months of age were associated with decreased subsequent *Giardia* detection (Supplementary Table 1). A combined 1 standard deviation greater zinc and retinol concentration was associated with an adjusted 22% (95% CI, 2%–37%) lower *Giardia*-detection rate in surveillance stool samples from 8 to 24 months of age. A higher retinol concentration at 15 months of age was also associated with an approximate 10% decrease in the subsequent *Giardia*-detection rate. There were no associations between zinc status and *Giardia* detection at 15 months of age and no associations at either time period between zinc or vitamin A status and incidence of *Giardia*-positive diarrhea.

*Giardia* detection in surveillance stool samples in the period between vitamin A status measurements at 7 and 15 months was associated with an adjusted −1.58 mg/dL (95% CI, −2.82 to −0.34 mg/dL) change in retinol concentration over that time period. There were no associations between *Giardia* and change in zinc concentration.

### *Giardia* and Risk of Acute Diarrhea

*Giardia* detection in surveillance stool samples was not associated with short-term diarrheal risk (adjusted RR for diarrhea in the following 30 days, 1.07 [95% CI, 0.96–1.19]). In addition, the apparent negative association between *Giardia* detection and diarrhea previously reported (RR adjusted for age and site, 0.90 [95% CI, 0.86–0.95]) [7] might be explained by treatment of 37% of all diarrhea episodes with metronidazole, such that *Giardia* might have been cleared before the diarrheal stool was collected. When we adjusted for recent metronidazole exposure, the association between *Giardia* detection and diarrhea moved toward the null (RR, 0.95 [95% CI, 0.90–1.00]).

*Giardia* detection was not associated with subsequent diarrheal rates in any except the PKN site (Supplementary Table 2). *Giardia* persistence before 6 months of age was common at the PKN site (17.4%) and was associated with an adjusted 28% relative decrease (95% CI, 11–41]) in diarrheal rates from 6 to 24 months of age. *Giardia* persistence in the first year of life at the PKN site (47.7%) was also associated with an adjusted decrease in subsequent diarrheal rates (adjusted incidence rate ratio, 0.81 [95% CI, 0.64–1.03]). The protective effect was driven largely by protection against subsequent diarrhea in which enteropathogenic bacteria were detected.

### Associations With Gut-Function Biomarkers

The presence of *Giardia* in stool samples was associated with an elevated marker of increased intestinal permeability, LMZ, across sites (Table 3), as indicated by an average increase in lactulose (z-score difference, 0.12 [95% CI, −0.00 to 0.24]) and a decrease in mannitol (z-score difference, −0.15 [95% CI, −0.26 to −0.04]). We found no significant differences in the associations across ages, although the magnitude was greatest in the second year of life (adjusted LMZ difference at 15 months, 0.25 [95% CI, 0.10–0.40]). Among markers of inflammation, *Giardia* was also associated with a decrease in NEO concentration (Table 3) but not consistently with MPO, ALA, or AGP.

**Table 3.** Associations Between *Giardia* Detection and Markers of Inflammation and Gut Permeability in All Stool Samples Among 2076 Children in the MAL-ED Cohort With at Least 1 Biomarker Measurement

Site	LMZ Difference^a^ (95% CI) (n = 4203)	ALA Difference^a^ (95% CI) (n = 24769)	MPO Difference^a^ (95% CI) (n = 24769)	NEO Difference^a^ (95% CI) (n = 24769)	AGP Difference^a^ (95% CI) (n = 3550)
BGD	0.29 (0.00 to 0.59)	0.1 (−0.06 to 0.27)	−0.22 (−0.43 to −0.02)	−0.07 (−0.35 to 0.21)	−0.44 (−12.01 to 11.14)
BRF	0.03 (−0.28 to 0.35)	0.00 (−0.22 to 0.23)	−0.18 (−0.46 to 0.09)	−0.20 (−0.42 to 0.02)	3.53 (−10.99 to 18.05)
INV	0.25 (0.04 to 0.46)	−0.08 (−0.2 to 0.03)	0.00 (−0.12 to 0.13)	−0.12 (−0.23 to −0.01)	−1.05 (−8.66 to 6.57)
NEB	0.18 (−0.15 to 0.51)	0.11 (−0.03 to 0.24)	0.01 (−0.14 to 0.16)	−0.10 (−0.21 to 0.01)	−1.05 (−11.62 to 9.51)
PEL	0.23 (0.07 to 0.39)	−0.02 (−0.16 to 0.11)	−0.12 (−0.25 to 0.02)	−0.19 (−0.30 to −0.08)	−5.59 (−15.91 to 4.74)
PKN	0.19 (0.01 to 0.38)	0.05 (−0.06 to 0.16)	0.00 (−0.11 to 0.10)	0.00 (−0.09 to 0.10)	7.39 (0.35 to 14.43)
SAV	0.17 (−0.27 to 0.61)	−0.01 (−0.19 to 0.18)	0.07 (−0.11 to 0.25)	−0.20 (−0.38 to −0.01)	4.45 (−11.05 to 19.94)
TZH	0.01 (−0.42 to 0.44)	0.01 (−0.13 to 0.15)	−0.06 (−0.19 to 0.08)	−0.23 (−0.39 to −0.07)	5.20 (−7.40 to 17.80)
All	0.22 (0.12 to 0.32)	−0.04 (−0.09 to 0.01)	−0.09 (−0.14 to −0.03)	−0.11 (−0.16 to −0.06)	1.83 (−1.82 to 5.47)

Abbreviations: AGP, α-1-acid glycoprotein (mg/dL); BGD, Dhaka, Bangladesh; BRF, Fortaleza, Brazil; CI, confidence interval; INV, Vellore, India; LMZ, urinary lactulose/mannitol excretion ratio z score (BRF cohort was the internal reference population); ALA, α-1-antitrypsin (log[mg/g]); MPO, myeloperoxidase (log[ng/mL]); NEB, Bhaktapur, Nepal; NEO: neopterin (log[nmol/L]); PEL, Loreto, Peru; PKN, Naushahro Feroze, Pakistan; SAV, Venda, South Africa; TZH, Haydom, Tanzania.

^a^Adjusted for site, age, stool consistency, sex, WAMI (Water, Assets, Maternal Education, Income) score, mother’s age, presence of siblings, water treatment, routine hand-washing after child defecation, use of toilet paper, dirt floor, ownership of chickens, percent exclusive breastfeeding, and presence of enteroaggregative *E coli* and/or *Campylobacter* in stool sample.

### Effects on Growth

*Giardia* detection was associated with reduced weight and length attainment at 2 years of age (Table 4). Compared with those with low *Giardia* exposure (the 10th percentile of *Giardia* positivity in surveillance stool samples over the first 2 years of life), children with high exposure (the 90th percentile of *Giardia* positivity) had an adjusted −0.12 LAZ decrement (95% CI, −0.25 to 0.01) and −0.11 WAZ decrement (95% CI, −0.23 to −0.00) at 2 years of age. *Giardia* persistence in the first 6 months of life was statistically significantly associated with more than double that decrement in both weight and length at 24 months of age (Table 4).

**Table 4.** Effects of *Giardia* Detection in Monthly Surveillance Stool Samples on Weight and Length Attainment at 2 Years of Age Among 1727 Children in the MAL-ED Cohort With Anthropometric Measurements at 2 Years

Exposure	n (N = 1727)	WAZ Difference^a^ (95% CI)	LAZ Difference^a^,^b^ (95% CI)
*Giardia* persistence in first 6 months	62	−0.29 (−0.53 to −0.05)	−0.29 (−0.64 to 0.07)
Any *Giardia* detection in first 6 months	173	−0.01 (−0.16 to 0.14)	0.08 (−0.12 to 0.28)
*Giardia* persistence in first year	290	−0.04 (−0.16 to 0.09)	−0.01 (−0.15 to 0.13)
Any *Giardia* detection in first year	549	−0.07 (−0.18 to 0.03)	−0.07 (−0.18 to 0.03)
*Giardia* persistence in second year	549	−0.05 (−0.15 to 0.05)	−0.09 (−0.19 to 0.01)
Any *Giardia* detection in second year	976	−0.01 (−0.11 to 0.08)	−0.05 (−0.14 to 0.05)
Any *Giardia* persistence	663	−0.05 (−0.15 to 0.04)	−0.07 (−0.17 to 0.03)
Any *Giardia* detection	1101	−0.02 (−0.12 to 0.08)	−0.04 (−0.13 to 0.06)
Any *Giardia*-related diarrhea^c^	557	0.07 (−0.04 to 0.19)	−0.03 (−0.15 to 0.10)
Percent positive surveillance stool samples (per 38% increase^d^)		−0.11 (−0.23 to −0.00)	−0.12 (−0.25 to 0.01)

Abbreviations: CI, confidence interval; LAZ, length-for-age z score; WAZ, weight-for-age z score.

^a^Adjusted for site, anthropometric measurement at enrollment, sex, WAMI (Water, Assets, Maternal Education, Income) score, mother’s age, presence of siblings, water treatment, routine hand-washing after child defecation, use of toilet paper, dirt floor, ownership of chickens, age at stopping exclusive breastfeeding, and percentage of surveillance stool samples that tested positive for *Campylobacter* and enteroaggregative *E coli* in the first 2 years of life.

^b^All LAZ estimates excluded PKN (n = 1478).

^c^Also adjusted for *Giardia* persistence in surveillance stool samples to assess independent effect of diarrhea with *Giardia* detection.

^d^The difference between the 10th and 90th percentile of cumulative percent positivity across all sites was 38%, which represents a contrast between high and low *Giardia* exposure in the context of the MAL-ED cohort.

## DISCUSSION

The prospective MAL-ED birth-cohort study provided a unique opportunity to investigate the complex relationships between *Giardia* exposure, micronutrient status, intestinal permeability, diarrhea, and growth. *Giardia* detection was common at all sites and increased in frequency through the second year of life. Major reductions in *Giardia* diarrhea incidence when more specific definitions were used suggest that true *Giardia*-caused cases of diarrhea are difficult to identify in settings of high endemicity. In the absence of molecular typing, evidence of visual clustering of *Giardia* detections within children and seasonal patterns limited to first detections suggest that repeated detections can often represent persistent infections. We identified both hygiene and environmental risk factors for *Giardia* infection and confirmed that the fecal-oral and waterborne routes both might be important modes of transmission. Host factors, including zinc and vitamin A deficiencies, might also contribute to *Giardia* susceptibility, because children with higher micronutrient concentrations had less subsequent *Giardia* detection. This relationship might be bidirectional, because *Giardia* detection from 7 to 15 months of age was also associated with a decrease in retinol concentration during that period.

In contrast to data from previous reports [8, 9], our longitudinal data did not suggest that *Giardia* infection was protective against diarrhea across the sites. The protective effect of *Giardia* on subsequent acute diarrheal risk was limited to early exposures at the PKN site, which might be explained by the uniquely high detection rate in the first year of life at this site, environment-specific unidentified biological susceptibility factors, *Giardia* strain variability, and coinfection factors. Because the diagnostic EIA was insensitive to stool consistency, dilution of *Giardia* in diarrheal stools does not explain previously reported inverse associations with diarrhea [4, 7]. In contrast, clearance of *Giardia* in diarrheal stools by metronidazole, the most common antibiotic used for diarrhea treatment in MAL-ED (17% of all episodes were treated with metronidazole [33]), might contribute to this inverse association.

Even in the absence of diarrheal symptoms, *Giardia* infection, especially early persistent infection, was associated with reduced weight and height attainment at 2 years. This finding was reported recently from an independent cohort study in Bangladesh [34]. This early impact might be the result of a critical period of susceptibility in which the infant gut and intestinal microbiota are developing [35, 36]. The increased lactulose/mannitol excretion ratio associated with *Giardia* suggests a mechanism through increased intestinal permeability and malabsorption, components of environmental enteropathy [18]. *Giardia* was not associated with increased markers of intestinal inflammation (ALA, MPO, or NEO), which suggests that *Giardia* might disrupt epithelial cells through pathways different from those of chronic immune activation typical of environmental enteropathy. Given the divergence between diarrhea and growth outcomes associated with *Giardia* infection, diarrhea-independent mechanisms are likely responsible for the growth impact such that physiologic insult occurs without necessary manifestation of diarrhea.

We found that the subset of children with early and persistent *Giardia* infection experienced a significant disease burden caused by *Giardia.* However, no association was observed between *Giardia* detection and growth in the comprehensive risk factor models from MAL-ED that compared high and low levels of *Giardia* exposures both defined by no detections in the first 6 months (MAL-ED Network Investigators, unpublished data). Although other determinants might influence growth at later ages more strongly, targeting *Giardia* early in life might confer substantial benefit.

This study was limited by fewer surveillance stool samples tested in the second year of life when *Giardia* was most common. In addition, although more sensitive than microscopy, the EIA has a lower sensitivity than nucleic acid–based detection methods [37, 38]. We also did not use Assemblage typing, which might have helped distinguish persistent infection from reinfection and explain variability in clinical outcomes [39, 40]. Last, antihelminthic medications that are active against *Giardia* (eg, albendazole or mebendazole) were not recorded, although they might have been used at the individual level and potentially through mass treatment campaigns. Despite these limitations, the breadth of data available, including markers of gut inflammation and permeability, enabled robust analysis of the effects and potential mechanisms of *Giardia* infection.

The MAL-ED study enabled a comprehensive description of *Giardia* epidemiology in children early in life across 8 diverse sites. In general, interventions to limit exposure might reduce *Giardia* burden better than treatment, because metronidazole only transiently reduced detection. Interventions to reduce early exposure (eg, through sustained exclusive breastfeeding) might have the greatest effect on long-term outcomes, given increased the susceptibility of children to the effects of *Giardia* during their first few months of life.

## Supplementary Data

Supplementary materials are available at the *Journal of the Pediatric Infectious Diseases Society* online.

## Supplementary Material

Supplemental_Material

## Notes

We thank the investigators and staff of the MAL-ED Network for their important contributions and the caregivers and children who participated in the study.

***Financial support.*** MAL-ED is carried out as a collaborative project supported by the Bill and Melinda Gates Foundation, the Foundation for the National Institutes of Health, and the National Institutes of Health Fogarty International Center (grant D43-TW009359 to E.T.R.).

***Potential conflicts of interest.*** All authors: No reported conflicts.
