# Supplementary material for: Determinants and Impact of Giardia Infection in the First 2 Years of Life in the MAL-ED Birth Cohort
Source: J Pediatric Infect Dis Soc. 2017 Feb 15;6(2):153–60. doi: 10.1093/jpids/piw082 (PMC5907871; doi:10.1093/jpids/piw082)
Supplement: Supplemental_Material [file piw082_suppl_supplemental_material.pdf]

Supplemental material

Determinants and impact of *Giardia* infection in the first two years of life in the MAL-ED birth cohort

Elizabeth T. Rogawski[1], Luther A. Bartelt[2], James A. Platts-Mills[1], Jessica C. Seidman[3], Amidou Samie[4], Alexandre Havt[5], Sudhir Babji[6], Dixner Rengifo Trigoso[7], Shahida Qureshi[8], Sadia Shakoor[8], Rashidul Haque[9], Estomih Mduma[10], Samita Bajracharya[11], S.M. Abdul Gaffar[9], Aldo A.M. Lima[5], Gagandeep Kang[6], Margaret N. Kosek[7,12], Tahmeed Ahmed[9], Erling Svensen[13], Carl Mason[14], Zulfiqar A Bhutta[8], Dennis R. Lang[15], Michael Gottlieb[15], Richard L. Guerrant[1], Eric R. Houpt[1], Pascal O. Bessong[4] and the MAL-ED Network Investigators

[1]Division of Infectious Diseases and International Health, University of Virginia, Charlottesville, [2]Division of Infectious Diseases, University of North Carolina-Chapel Hill, [3]Fogarty International Center, National Institutes of Health, Bethesda, [4]University of Venda, Thohoyandou, South Africa, [5]Clinical Research Unit and Institute of Biomedicine, Federal University of Ceara, Fortaleza, Brazil, [6]Christian Medical College, Vellore, India, [7]Asociación Benéfica PRISMA, Iquitos, Peru, [8]Aga Khan University, Karachi, Pakistan, [9]International Centre for Diarrhoeal Disease Research, Dhaka, Bangladesh, [10]Haydom Lutheran Hospital, Haydom, Tanzania, [11]Walter Reed AFRIMS Research Unit Nepal, Kathmandu, Nepal, [12]Bloomberg School of Public Health, Johns Hopkins University, Baltimore, [13]Haukeland University Hospital, Bergen, Norway, [14]Armed Forces Research Institute of Medical Sciences, Bangkok, Thailand, [15]Foundation for the National Institutes of Health, Bethesda

MAL-ED Investigators and Institutional Affiliations

Angel Mendez Acosta[1], Rosa Rios de Burga[1], Cesar Banda Chavez[1], Julian Torres Flores[1], Maribel Paredes Olotegui[1], Silvia Rengifo Pinedo[1], Mery Siguas Salas[1], Dixner Rengifo Trigoso[1], Angel Orbe Vasquez[1], Imran Ahmed[2], Didar Alam[2], Asad Ali[2], Zulfiqar A Bhutta[2], Shahida Qureshi[2], Muneera Rasheed[2], Sajid Soofi[2], Ali Turab[2], Anita KM Zaidi[2,] Ladaporn Bodhidatta[3], Carl J Mason[3], Sudhir Babji[4], Anuradha Bose[4], Ajila T George[4], Dinesh Hariraju[4], M. Steffi Jennifer[4], Sushil John[4], Shiny Kaki[4], Gagandeep Kang[4], Priyadarshani Karunakaran[4], Beena Koshy[4], Robin P Lazarus[4], Jayaprakash Muliyil[4], Mohan Venkata Raghava[4], Sophy Raju[4], Anup Ramachandran[4], Rakhi Ramadas[4], Karthikeyan Ramanujam[4], Anuradha Rose[4], Reeba Roshan[4], Srujan L Sharma[4], Shanmuga Sundaram E[4], Rahul J Thomas[4], William K Pan[5,6], Ramya Ambikapathi[6], J Daniel Carreon[6], Vivek Charu[6], Viyada Doan[6], Jhanelle Graham[6], Christel Hoest[6], Stacey Knobler[6], Dennis R Lang[6, 7], Benjamin JJ McCormick[6], Monica McGrath[6], Mark A Miller[6], Archana Mohale[6,] Gaurvika Nayyar[6], Stephanie Psaki[6], Zeba Rasmussen[6], Stephanie A Richard[6], Jessica C Seidman[6], Vivian Wang[6], Rebecca Blank[7], Michael Gottlieb[7], Karen H Tountas[7], Caroline Amour[8], Eliwaza Bayyo[8], Estomih R Mduma[8], Regisiana Mvungi[8], Rosemary Nshama[8], John Pascal[8], Buliga Mujaga Swema[8], Ladislaus Yarrot[8], Tahmeed Ahmed[9], AM Shamsir Ahmed[9], Rashidul Haque[9], Iqbal Hossain[9], Munirul Islam[9], Mustafa Mahfuz[9], Dinesh Mondal[9], Fahmida Tofail[9], Ram Krishna Chandyo[10], Prakash Sunder Shrestha[10], Rita Shrestha[10], Manjeswori Ulak[10], Aubrey Bauck[11], Robert Black[11], Laura E Caulfield[11], William Checkley[11,6], Margaret N Kosek[11], Gwenyth Lee[11], Kerry Schulze[11], Pablo Peñataro Yori[11], Laura E. Murray-Kolb[12], A Catharine Ross[12], Barbara Schaefer[12,6], Suzanne Simons[12], Laura Pendergast[13], Cláudia B Abreu[14], Hilda Costa[14], Alessandra Di Moura[14], José Quirino Filho[14,6], Alexandre Havt[14], Álvaro M Leite[14], Aldo AM Lima[14], Noélia L Lima[14], Ila F Lima[14], Bruna LL Maciel[14], Pedro HQS Medeiros[14], Milena Moraes[14], Francisco S Mota[14], Reinaldo B Oriá[14,] Josiane Quetz[14], Alberto M Soares[14],Rosa MS Mota[14], Crystal L Patil[16], Pascal Bessong[17], Cloupas Mahopo[17], Angelina Maphula[17], Emanuel Nyathi[17], Amidou Samie[17], Leah Barrett[18], Rebecca Dillingham[18], Jean Gratz[18], Richard L Guerrant[18], Eric Houpt[18], William A Petri, Jr[18], James Platts-Mills[18], Rebecca Scharf[18], Elizabeth T. Rogawski[18], Binob Shrestha[19], Sanjaya Kumar Shrestha[19], Tor Strand[19,15], Erling Svensen[20,8]

[1]A.B. PRISMA, Iquitos, Peru, [2]Aga Khan University, Karachi, Pakistan, [3]Armed Forces Research Institute of Medical Sciences, Bangkok, Thailand, [4]Christian Medical College, Vellore, India, [5]Duke University, Durham, NC, USA, [6]Fogarty International Center/National Institutes of Health, Bethesda, MD, USA, [7]Foundation for the NIH, Bethesda, MD, USA, [8]Haydom Lutheran Hospital, Haydom, Tanzania, [9]icddr,b, Dhaka, Bangladesh, [10]Institute of Medicine, Tribhuvan University, Kathmandu, Nepal, [11]Johns Hopkins University, Baltimore, MD, USA, [12]The Pennsylvania State University, University Park, PA, USA, [13]Temple University, Philadelphia, PA, USA, [14]Universidade Federal do Ceara, Fortaleza, Brazil, [15]University of Bergen, Norway, [16]University of Illinois at Chicago, IL, USA, [17]University of Venda, Thohoyandou, South Africa, [18]University of Virginia, Charlottesville, VA, USA, [19]Walter Reed/AFRIMS Research Unit, Kathmandu, Nepal, [20]Haukeland University Hospital, Bergen, Norway

Figure S1. Heat maps showing *Giardia* positivity in surveillance stools over the first 2 years of life among children in the MAL-ED cohort with more than one *Giardia*-positive stool in Bangladesh, India, Nepal and Pakistan (n=444 of 1016 total children in the South Asian sites; A) and in Brazil, Peru, Tanzania, and South Africa (n=394 of 1072 total children in the southern hemisphere sites; B). Each row represents positivity in the stools of one child; children are ordered by detection frequency.

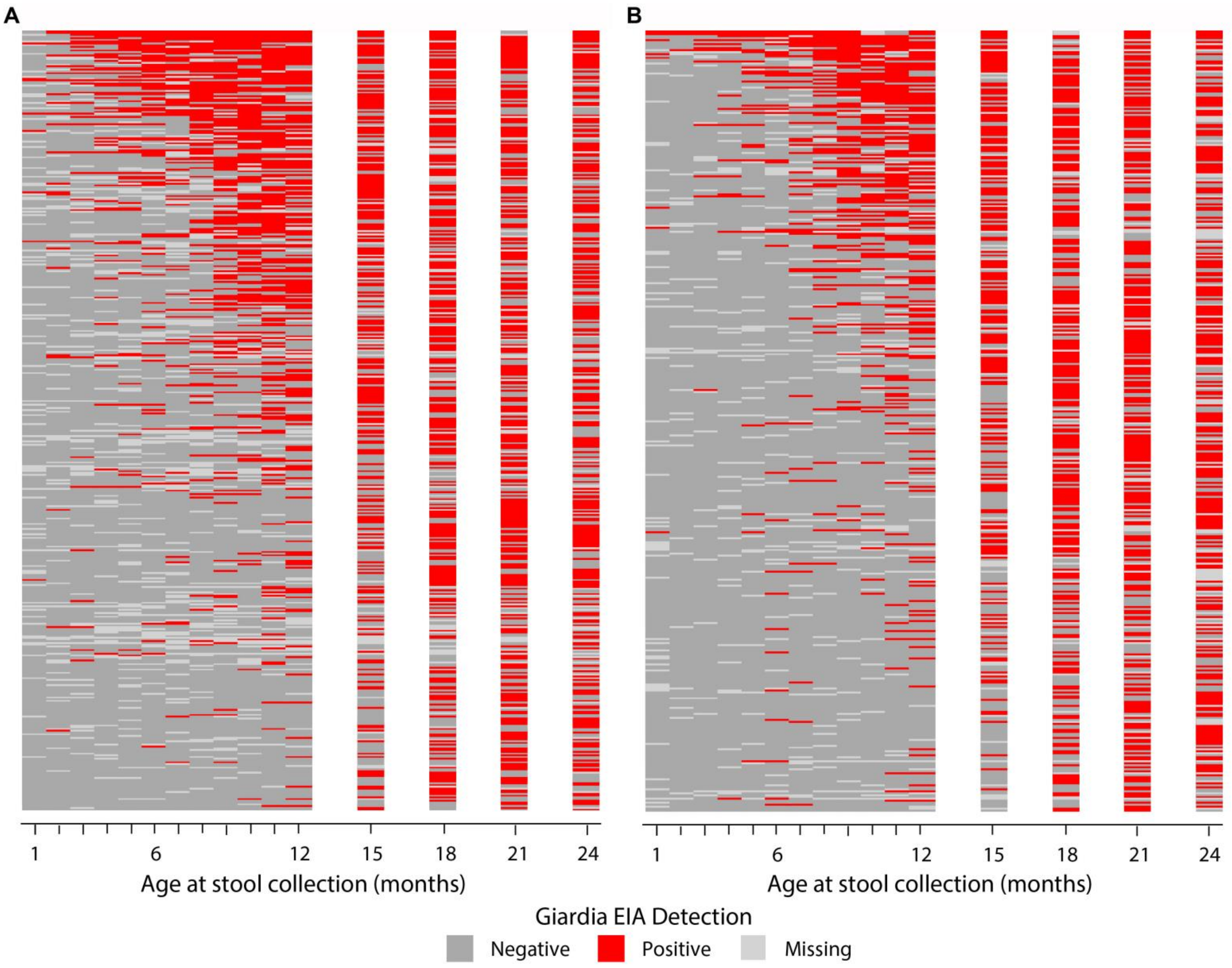

Figure S2. Site-specific estimates of key risk factors for first *Giardia* detection in monthly surveillance stools among 2,088 children in the MAL-ED cohort with at least one surveillance stool.

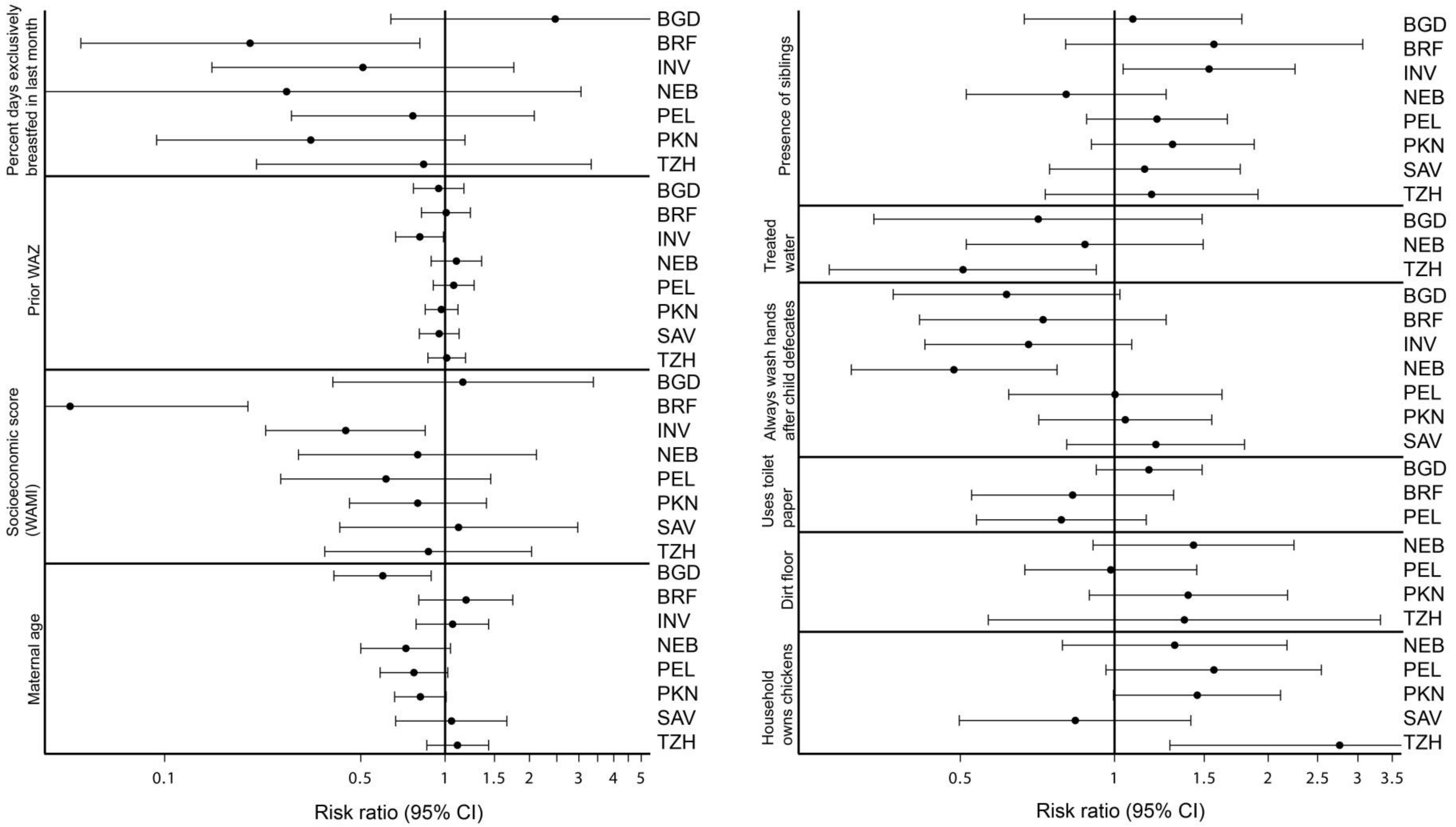

BGD – Dhaka, Bangladesh; BRF – Fortaleza, Brazil; INV – Vellore, India; NEB – Bhaktapur, Nepal; PEL – Loreto, Peru; PKN – Naushahro Feroze, Pakistan; SAV – Venda, South Africa; TZH – Haydom, Tanzania

Figure S3. Incidence of first *Giardia* detection in surveillance stools among 2,088 children in the MAL-ED cohort over months of the year for the **A**: South Asian sites (Bangladesh, India, Nepal, and Pakistan); and **B**: southern hemisphere sites (Brazil, Peru, Tanzania, and South Africa).

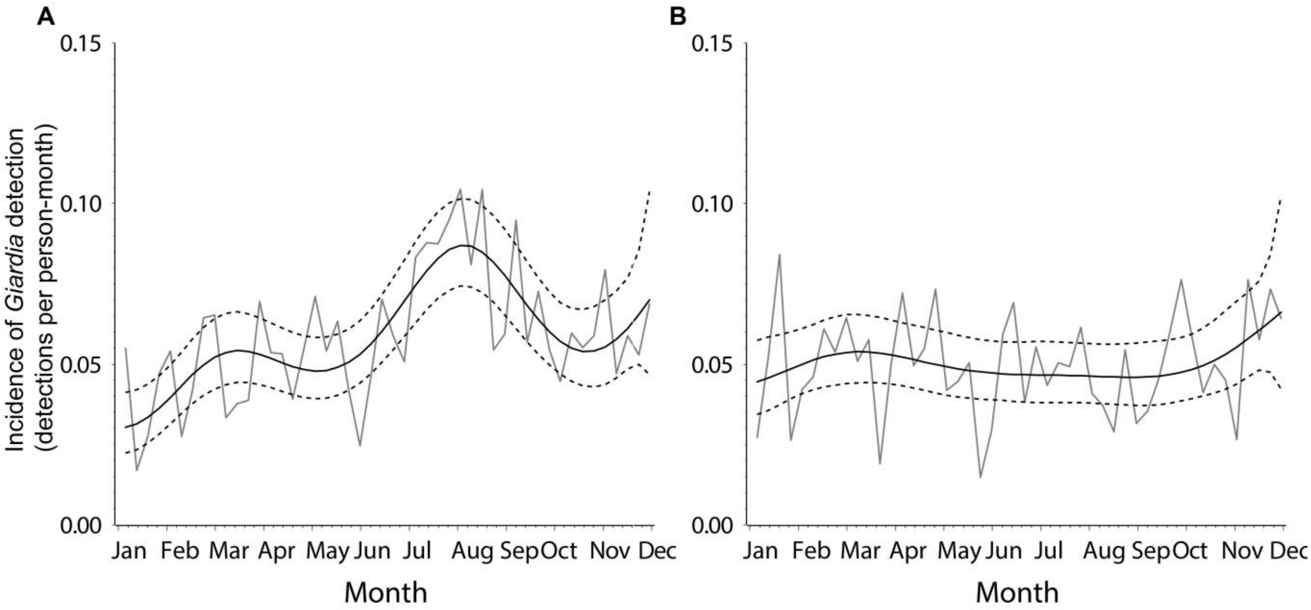

Table S1. Associations between micronutrient status and *Giardia* detection in surveillance stools among 1,521 children in the MAL-ED cohort with an assessment of plasma zinc and retinol concentration at 7 months of age.

Micronutrient	Detection rate ratio* (95% CI)
Zinc concentration (per 50 mcg/dL†)	0.81 (0.67, 0.97)
Retinol concentration (per 10 mcg/dL†)	0.89 (0.79, 1.01)
Both higher zinc (50 mcg/dL) and retinol (10 mcg/dL) concentrations	0.78 (0.63, 0.98)

*Rate ratio of *Giardia* detections in surveillance stools from 8 to 24 months of age, adjusted for site, *Giardia* detection prior to blood draw, enrollment weight, sex, WAMI, mother's age, presence of siblings, water treatment, routine handwashing after child defecation, use of toilet paper, dirt floor, ownership of chickens and proportion of days exclusively breastfed in month before blood draw. Analysis excludes the South African site.

†Approximately the standard deviation of the micronutrient in the study population.

Table S2. Effect of early *Giardia* presence and persistence on subsequent all-cause diarrheal rates in Naushahro Feroze, Pakistan and all other sites among 1,967 children in the MAL-ED cohort who remained in the study for at least 6 months.

	Naushahro Feroze, Pakistan (n=265)			All other sites (n=1702)		
Giardia exposure	No. exposed	aIRR* (95% CI)	*p*-value	No. exposed	aIRR* (95% CI)	*p*-value
Persistence in first 6 months	46	0.72 (0.59, 0.89)	0.002	27	1.13 (0.78, 1.64)	0.5
Any detection in first 6 months	106	0.79 (0.66, 0.95)	0.01	88	1.19 (1.00, 1.41)	0.06
Persistence in first year	122	0.81 (0.64, 1.03)	0.09	188	1.12 (0.96, 1.29)	0.1
Any detection in first year	194	1.06 (0.81, 1.38)	0.7	388	1.10 (0.98, 1.23)	0.1
Persistence in second year	132	0.75 (0.55, 1.01)	0.06	436	1.00 (0.86, 1.18)	0.9
Any detection in second year	210	0.84 (0.60, 1.18)	0.3	806	1.09 (0.94, 1.26)	0.3

*****Incidence rate ratio for diarrhea following exposure period (after 6 months, 12 months, or 18 months), adjusted for site, age, sex, socioeconomic score (WAMI), mother's age, presence of siblings, water treatment, routine handwashing after child defecation, use of toilet paper, dirt floor, ownership of chickens, and days with diarrhea, acute lower respiratory infection, fever, and vomiting in exposure period
